# Case Report: Effect of transcutaneous auricular vagus nerve stimulation on acute pain after external fixation for bilateral open tibiofibular fractures

**DOI:** 10.3389/fnins.2025.1586632

**Published:** 2025-07-03

**Authors:** Qi Li, Donglin Zhao, Shuyang Zhang, Xinyu Du, Ning Ding, Zheng Xing, Xiaolei Chu, Weiguo Xu

**Affiliations:** ^1^Department of Rehabilitation, Tianjin University Tianjin Hospital, Tianjin, China; ^2^Tianjin Key Laboratory of Exercise Physiology and Sports Medicine, Institute of Sport, Exercise and Health, Tianjin University of Sport, Tianjin, China

**Keywords:** postoperative tibiofibular fracture, acute pain, transcutaneous auricular vagus nerve electrical stimulation, analgesia, pain mangement

## Abstract

A 50-year-old female patient sustained bilateral tibiofibular fractures due to a traffic accident and underwent open external fixation surgery on both lower limbs. Postoperatively, she reported severe pain (VAS ≥ 8). The patient had no significant underlying medical conditions or contraindications to vagal nerve stimulation. Transcutaneous auricular vagus nerve stimulation (taVNS)—a non-invasive modality that activates vagal pathways to modulate sympathetic-parasympathetic balance, suppress inflammation, and inhibit pain signaling—was trialed as an adjunctive analgesic intervention. Heart rate variability (HRV) was incorporated as an objective biomarker to quantify dynamic changes in autonomic function and their association with pain relief. After stabilization, taVNS was administered continuously for two weeks. Parameters monitored included immediate pressure pain thresholds (PPT) during intervention, daily postoperative analgesic consumption, and pre-/post-intervention Visual Analog Scale (VAS) and Generalized Anxiety Disorder-7 (GAD-7) scores. Dynamic electrocardiography was used to record HRV parameters (HR, SDNN, RMSSD, LF, HF, LF/HF). Post-intervention, the patient demonstrated significant reductions in VAS scores, a progressive increase in PPT, and alleviated anxiety. HRV analysis revealed enhanced parasympathetic activity and improved sympathovagal balance. This case suggests that taVNS may effectively alleviate acute postoperative pain by modulating autonomic function. Dynamic HRV monitoring provided objective evidence of pain-autonomic nervous system interactions, supporting taVNS as a complementary strategy for postoperative pain management. However, as a single-case report, this study has limited sample size, necessitating further large-scale randomized controlled trials to validate these findings.

## 1 Introduction

Transcutaneous auricular vagus nerve stimulation (taVNS) is a safe, non-invasive, portable, non-invasive method of electrical stimulation that modulates the autonomic nervous system (ANS) via stimulation of the vagus nerve. taVNS modulates ANS function primarily through central projections and cholinergic anti-inflammatory pathways to restore or reduce perioperative autonomic dysregulation and thereby reduce pain and stress (Kaniusas et al., 2019). TaVNS is widely utilized in the treatment of refractory epilepsy and treatment-resistant depression. Due to its non-invasive nature, ease of integration into clinical workflows, and high patient compliance, it has become a preferred therapeutic option in these areas.

Tibiofibular fractures are categorized as traumatic fractures that primarily result from road traffic accidents and falls. It is a prevalent clinical lower limb fracture. External fixation is a well-established treatment modality for traumatic tibiofibular fractures, offering the benefits of minimal trauma and a favorable prognosis. Subsequent to external fixation, the region of tissue damage activates injury receptors and non-neural cells, leading to the release of substantial inflammatory mediators (Ackland et al., 2019). Furthermore, the postoperative patient’s diminished capacity to undertake day-to-day activities, in conjunction with apprehensions pertaining to the functional rehabilitation of the lower limbs, renders them susceptible to psychological distress, manifesting as anxiety and depression. In patients exhibiting postoperative anxiety, the activation of noradrenergic neurons within the NAcc triggers changes in the ANS (Kendler et al., 1992), which manifests itself as sympathetic-adrenomedullary and cardiovascular hyperreactivity, with weakened vagal counterbalance to sympathetic action, a slowed heart rate, and elevated blood pressure, which further exacerbates pain (Kjellgren and Gomes, 1993).

Research indicates that taVNS may effectively mitigate acute pain following orthopedic procedures and decrease reliance on postoperative opioid analgesics (Patel et al., 2023). However, this study has certain limitations. Due to the broad inclusion criteria of patients, it lacks specificity in addressing acute pain following a single type of orthopedic surgery. Therefore, the present research aims to investigate the effects of taVNS on acute pain after external fixation surgery for bilateral tibiofibular fractures, thereby providing practical and theoretical support for clinical practice.

### 1.1 History and diagnostics

The patient is a 50-year-old female of Han ethnicity. In September 2024, she sustained injuries in a traffic accident resulting in pain, bleeding, and deformity in the lower back, sacrococcygeal region, and distal ends of both lower legs, accompanied by restricted mobility. There was no numbness or weakness (neuropathic symptoms) in either lower limb. X-rays obtained upon hospital admission revealed: Bilateral distal tibiofibular fractures, L1 vertebral burst fracture, L1 spinous process fracture, S3-S5 fractures, and a right 12th rib fracture. The diagnosis was established. Comprehensive evaluation, including laboratory tests, physical examination, and imaging studies, revealed no absolute contraindications for surgery. Consequently, on September 29th, the patient underwent the following procedures under emergency nerve block anesthesia: Open reduction and unilateral external fixation for the open left tibiofibular fracture, and open reduction and circular external fixation for the open right tibiofibular fracture. Additionally, the patient has an L1 vertebral burst fracture (ASIA: Grade E) and S3-S4 fractures. For these spinal injuries, she is currently undergoing conservative treatment, which includes strict bed rest and external brace fixation. The patient was previously healthy with no significant medical, familial, psychosocial, or genetic history.

The patient’s overall condition was stable after surgery, with a positive healing trend at the surgical incision site. They were transferred to the Rehabilitation Department on October 10th for systematic rehabilitation therapy. Physical examination: The patient is in the supine position; external fixation brackets are visible on both lower legs. A scratch approximately 2 cm long is visible on the distal left lower leg, and an irregular wound, approximately 6 cm long, is present on the distal right lower leg. The wounds showed good healing, without significant redness, swelling, or exudation; the sutures had not been removed. Range of motion in both hip and knee joints was limited. Muscle strength was grade 3 on the left side and grade 2 on the right side. Range of motion and muscle strength in both upper limbs showed no significant abnormalities, sensation was normal, and pedal pulses were palpable. X-ray (radiograph) results showed status post external fixation surgery for comminuted fractures of the distal segments of the left and right tibia and fibula. Pain intensity was assessed using the Numerical Rating Scale (VAS), with the patient reporting VAS scores > 8 postoperatively. Therefore, we selected a portable, low-cost, safe, and effective neuromodulation technology to regulate the Autonomic Nervous System (ANS) for the purpose of alleviating postoperative pain. Prior to the commencement of the experiment, the patient underwent a comprehensive clinical assessment with no contraindications identified.

### 1.2 Primary drug therapy

On admission, the patient was educated about pain, and patients with insomnia and anxiety could be given appropriate oral sleep aids and antianxiety medication. The patient was routinely given 0.8 g of ibuprofen (Chengdu Bite Pharmaceutical Co., Ltd., State Drug License H20203474) released in a 250 ml intravenous 0.9% sodium chloride solution (Sichuan Kelun Pharmaceutical Co., Ltd., State Drug License H51021157) for 3 days after surgery. In combination with oral analgesics, analgesics may be discontinued if the VAS score is < 3.

### 1.3 Transcutaneous auricular vagus nerve stimulation

The electrical spikes of a transcutaneous auricular vagus nerve electrical stimulator (Beijing Feiyuxing Electronic Technology Co., Ltd.) were placed on the patient’s left ear in the auricular submarine, which is completely innervated by the vagus nerve (Butt et al., 2020). The stimulation parameters were determined according to a systematic review (Patel et al., 2022): The stimulation frequency was 30 Hz, the pulse width was 200 μs, the stimulation time was 30, 30 s of interruption, and the cycle was repeated for 30 min once a day for two weeks. The stimulation intensity was such that the patient could feel tingling but not pain or discomfort.

### 1.4 Nursing records

Vital signs (including body temperature, pulse, blood pressure, and respiratory rate) were recorded over the two-week intervention period. Additionally, the patient’s daily bowel movement frequency was documented (Table 1).

**TABLE 1:** Patient care record.

	10.16	10.17	10.18	10.19	10.20	10.21	10.22	10.23	10.24	10.25	10.26	10.27
Body temperature (°C)	36.6	36.4	36.6	36.2	36.3	36.5	36.2	36.6	36.3	36.3	36.6	36.5
Pluse (time/min)	68	76	75	73	74	77	72	66	79	74	74	68
Blood pressure (mmHg)	98/57	/	/	/	/	/	98/57	/	/	/	/	/
Breathe (time/min)	18	17	16	17	19	18	19	18	18	16	18	18
Defecating frequency (time/day)	1	1	1	1	1	1	1	1	1	1	1	1

## 2 Evaluation of indicators

### 2.1 Visual Analog Scale

Postoperative subjective pain was assessed using a 0–10 numerical rating scale, where 0 indicates “no pain” and 10 represents “the most intense unbearable pain.”

### 2.2 Pressure pain threshold

The PPT was measured using a Baseline visual pain measurement device. Referencing anatomical landmark guidelines (In et al., 2024), the 1 cm^2^ probe was applied perpendicularly with uniform pressure at a point 10 cm distal to the medial humeral epicondyle. This location corresponds to the midpoint of the line connecting the medial humeral epicondyle to the ulnar styloid process. The measurement was halted when the subject first perceived pain. This procedure was repeated three times, with the mean value calculated from the readings.

### 2.3 Generalized Anxiety Disorder-7 (GAD-7)

This 7-item questionnaire assesses anxiety severity, with each item scored on a 0−3 scale. Total scores range from 0 to 21: 0−4 (normal anxiety), 5−9 (mild anxiety), 10−13 (moderate anxiety), 14−18 (moderately severe anxiety), and 19−21 (severe anxiety).

### 2.4 Heart rate variability

**FIGURE 1:**
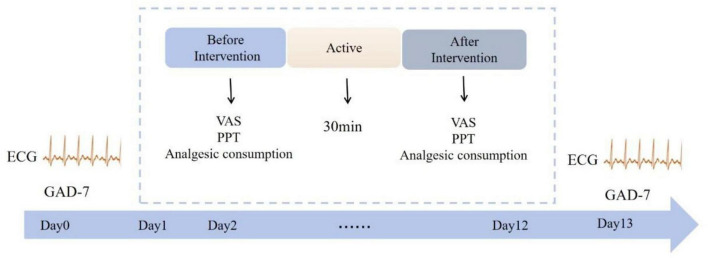
Specific experimental process. Patient electrocardiograms and GAD-7 scores were monitored at baseline (day 0) and post-intervention (day 13). The intervention spanned 2 weeks (days 1–12), with VAS scores, PPT values, and analgesic consumption recorded before and after each session. Electrical stimulation was administered once daily for 30 min per session.

ECG signals (sampling rate: 200 Hz) were recorded for 5 min pre- and post-intervention using a Healink electrocardiograph (Bengbu Hailian Health Management Center Co., Ltd., China) with a V5 chest lead configuration. Electrodes were placed at the right mid-clavicular line (first intercostal space) and left anterior axillary line (fourth intercostal space). Participants rested for 2 min before recording in a quiet laboratory (25–27°C). Experimental details are shown in Figure 1.

HRV analysis: RR intervals were extracted using MATLAB (The Mathworks Inc., United States). Time-domain parameters (mean HR, SDNN, RMSSD) and frequency-domain parameters (LF, HF, LF/HF ratio) were calculated. For power spectral analysis, RR intervals were resampled at 4 Hz using spline interpolation. Fast Fourier Transform generated power spectral density (PSD)(Pomeranz et al., 1985),with LF (0.04–0.15 Hz) and HF (0.15–0.4 Hz) frequency bands.

## 3 Results

**FIGURE 2:**
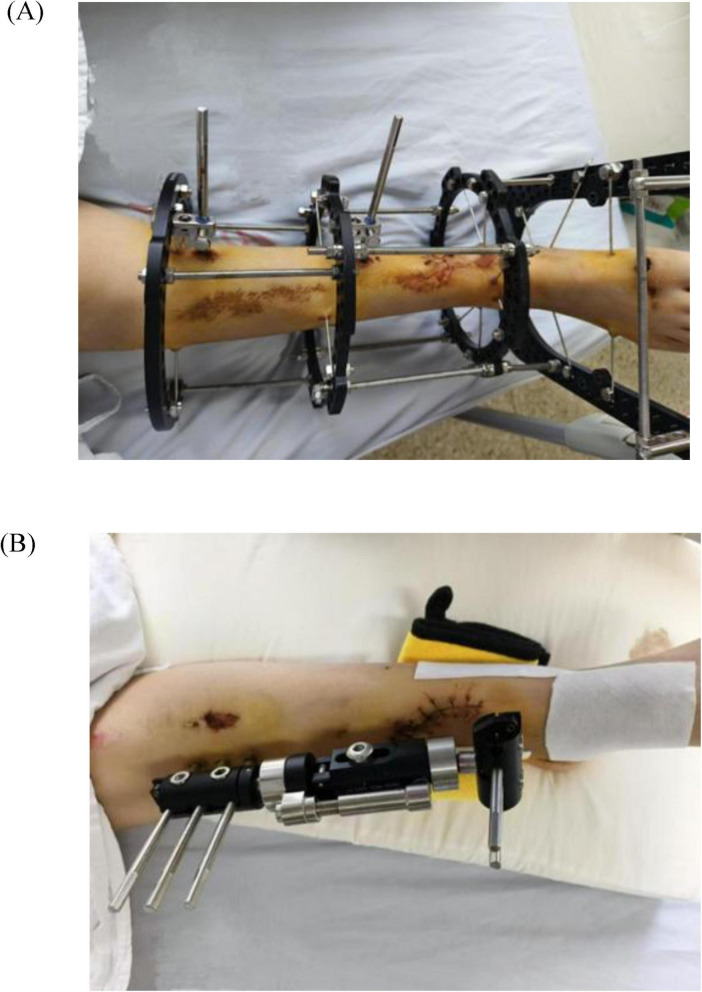
**(A)** Right tibiofibular fracture after external fixation. **(B)** Left tibiofibular fracture after external fixation.

**FIGURE 3:**
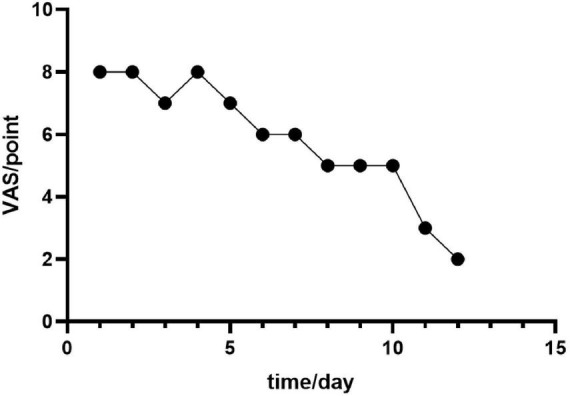
VAS score before and after intervention. BI, before intervention; AI, after intervention.

The study reports findings from a two-week investigation. Simple linear regression analysis revealed a statistically significant negative association between intervention duration (days) and the magnitude of VAS score reduction (β = −0.946, *p* < 0.001). For each additional day of treatment, the VAS score decreased by a mean of 0.946 points (95% CI: 4.60−7.07 reduction). This model indicates that intervention duration explained 89.4% of the variability in VAS scores (*R*^2^ = 0.894). Despite the negative correlation between time and VAS scores, the observed reduction was less than the minimal clinically important difference (MCID = 2 points for clinicians), suggesting the effect may lack clinical significance (Figure 2). This outcome may be attributable to the inherent subjectivity and measurement variability associated with VAS scores (Figure 3).

**FIGURE 4:**
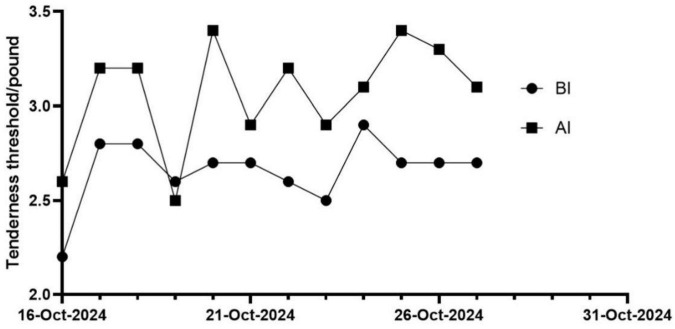
PPTs before and after intervention. BI, before intervention; AI, after intervention.

The changes in patients’ PPT immediately after each treatment session were analyzed. The results showed that compared to pre-intervention, post-intervention PPT values demonstrated a gradual increasing trend (Figure 4).

**FIGURE 5:**
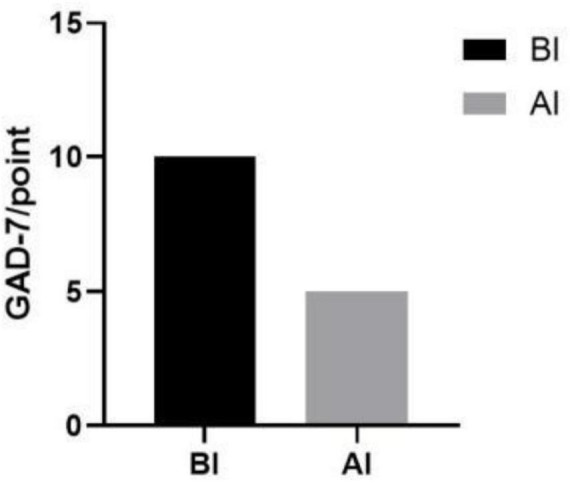
Results of anxiety scores before and after the intervention. BI, before intervention; AI, after intervention.

Furthermore, a decrease in patient anxiety levels was observed postintervention compared with the preintervention period, with mild anxiety levels reported (Figure 5).

**FIGURE 6:**
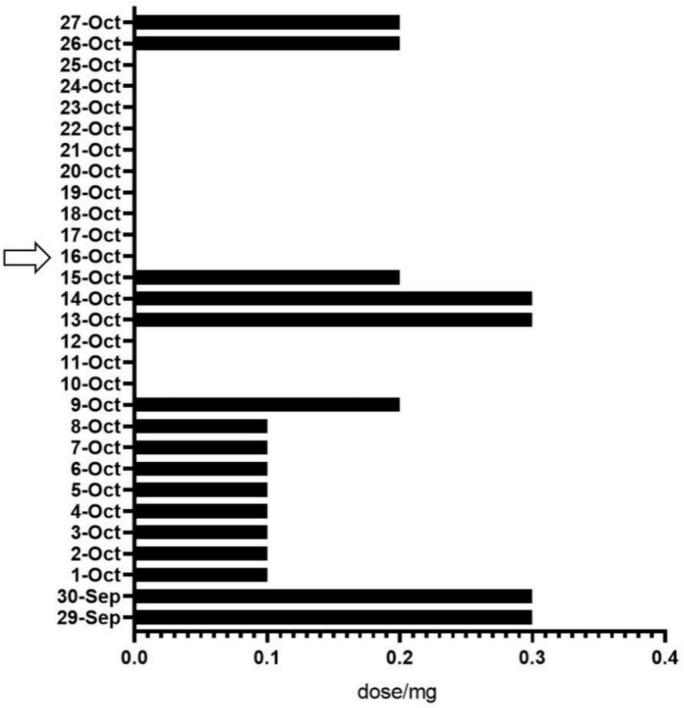
Analgesic drug use before and after the intervention. The arrows represent the start of the intervention.

After surgery, patients received analgesic therapy with NSAIDs. The results showed that, compared to the period from the first postoperative day to pre-intervention (average dosage: 135.3 mg), the analgesic medication usage was significantly reduced in patients after the intervention (average dosage: 33.3 mg) (Figure 6).

The Holter data capture rate for autonomic analyses during taVNS was 100%. For the time-domain metrics, a decrease in the mean heart rate was observed, whereas the SDNN and RMSSD were significantly greater after the intervention than before the intervention. For the frequency domain metrics, LF, HF power, and LF/HF were also significantly elevated (Table 2).

**TABLE 2:** HRV changes before and after interventiom.

	HR/time	SDNN/ms	RMSSD/ms	LF/ms^2^	HF/ms^2^	LF/HF
Before intervention	65	16.2	9	28	27	1.06
After intervention	57	23	12.3	90	54	1.657

## 4 Conclusion and discussion

This case report describes the effects of transcutaneous auricular vagus nerve stimulation (taVNS) on acute postoperative pain in a patient following open reduction and external fixation for bilateral tibiofibular fractures. During the two-week taVNS intervention, results indicate that active peripheral neuromodulation via the auricular nerve may alleviate postoperative pain and anxiety, decrease pain sensitivity, and reduce analgesic requirements.

In experimental studies, noxious stimulation was administered to healthy individuals, revealing that changes in the LF/HF ratio during taVNS exhibited a negative correlation with the nociceptive withdrawal threshold. This suggests a progressive increase in parasympathetic nervous activity during treatment (Yokota et al., 2024). When applying transcutaneous auricular vagus nerve stimulation (taVNS) to manage postoperative pain in orthopedic surgery, it was found that taVNS not only alleviated pain and reduced opioid consumption, but also significantly decreased sympathetic nerve activity (as indicated by reductions in SDNN and LF), with observed changes exceeding the minimum clinically important difference (MCID) (Patel et al., 2023). In experiments where healthy adults were subjected to cold stimulation at different time intervals, particularly following sustained cold stimulation before taVNS administration, RMSSD (root mean square of successive differences) was found to induce a significant positive increase, indicating marked enhancement of parasympathetic nervous activity (Ehrens et al., 2024). To further investigate its effects on specific orthopedic postoperative pain, Zhou et al. (2022) conducted a study on patients undergoing anterior cruciate ligament (ACL) reconstruction. They found that taVNS not only reduced the incidence and duration of postoperative rebound pain and the frequency of patient-controlled analgesia (PCA) requests but also markedly improved pain-related sleep disturbances. Additionally, taVNS showed positive outcomes in perianal postoperative pain management. Compared to sham stimulation, taVNS significantly lowered VAS during wound dressing changes and defecation on the first postoperative day, decreased analgesic requirements, reduced urinary retention incidence, and demonstrated excellent patient tolerability (Yin et al., 2025). In summary, taVNS exhibits broad and promising therapeutic potential for perioperative pain management.

Inflammation is a complex process that is regulated through interconnected humoral and neuroreflex pathways. The vagus nerve plays a significant role in the neuroendocrine-immune axis, which not only regulates AMS function but also modulates inflammation. Furthermore, both invasive (Namgung et al., 2022) and non-invasive electrical (Addorisio et al., 2019) stimulation of the vagus nerve significantly suppresses cellular inflammatory responses. taVNS forms the ear-vagus sensory pathway by recruiting sensory fibers from the vagus nerve and projecting them to the brainstem for neuromodulation. The primary anti-inflammatory reflex pathways associated with the vagus nerve include the hypothalamic–pituitary–adrenal axis. Vagal afferent fibers are responsible for sensing the extent and location of injury, whereas inflammatory cytokines activate the solitary tract nucleus in response to the activation of vagal nerve endings. This, in turn, activates specific neurons in the hypothalamus, leading to the release of adrenocorticotropic hormone from the pituitary gland. This, in turn, stimulates the release of glucocorticoids from the adrenal cortex, thereby reducing peripheral inflammation (Kaniusas et al., 2019). (2) The anti-inflammatory vagal reflex pathway, also known as the cholinergic anti-inflammatory pathway, is initiated by the activation of afferent and efferent vagal neurons during the process of inflammation. This results in the release of acetylcholine from their synaptic terminals, which bind to the α-7 nicotinic-type receptors present on macrophages. This binding serves to downregulate the expression of inflammatory factors, such as tumor necrosis factor α, interleukin (IL)-1β and IL-6, thereby reducing pain sensitivity (Namgung et al., 2022). Furthermore, preliminary studies have demonstrated that momentary electrical stimulation of the vagus nerve initiates an anti-inflammatory response with a duration of more than 24 h (Olofsson et al., 2015). Modulation of the vagus nerve, resulting in a reduction in proinflammatory cytokines and an increase in anti-inflammatory cytokines, has been shown to play a pivotal role in the suppression of excessive inflammation, prevention of tissue damage, and enhancement of norepinephrine levels.

In addition to activating anti-inflammatory pathways, taVNS also provides indirect analgesia through the activation of gate control. taVNS activates the downstream inhibitory system by stimulating Aβ fibers in the ear that project through the nucleus tractus solitarius to other brainstem complexes (interacting with spinal cord regions involved in pain processing), which attenuates nociceptive signals traveling downstream into the gray matter of the dorsal horn of the spinal cord and reduces pain perception (Ellrich and Lamp, 2005). Furthermore, taVNS has been demonstrated to facilitate chemical modulation, thereby impeding the transmission of nociceptive signals to the brain by augmenting the release of endogenous opioid peptides and various neurotransmitters (e.g., enkephalins and substance P) within the central nervous system (Lehtimäki et al., 2013). Increased chemical modulation of the release of serotonin, norepinephrine, and endogenous opioid peptides in the brain has been associated with nociceptive processing and mood.

In contrast to the prevailing view that pressure pain sensitivity merely reflects the activity of peripheral nociceptors arising in deep tissues, it is now recognized that it also reflects the excitability of secondary neurons in the spinal cord. A study of healthy women revealed that the temporal summation of afferent neural signals due to mechanically induced pressure muscle pain was more pronounced than that due to skin pain stimuli (Nie et al., 2005). Furthermore, Busch et al.(2013) demonstrated that the PPT increased and decreased pain ratings and temperature pain thresholds after taVNS, suggesting that subjects had reduced involvement in central zone pain processing. The results of the present study were similar to those of that study, suggesting that taVNS may affect central pain processing and reduce central excitability. In addition to increasing pressure pain thresholds, it has been demonstrated that taVNS also increases electrical pain thresholds by 30–50% (Busch et al., 2013).

The role of the vagus nerve in mood regulation is well documented. Stimulation of the vagus nerve by taVNS has been shown to modulate neurotransmitter activity, including serotonin and adrenaline, which are critical for mood regulation. According to functional magnetic resonance imaging (fMRI) and neural tracing methods, taVNS activates the solitary tract nucleus, the blue spot, the trigeminal spinal tract nucleus, the cuneate row nucleus, the dorsal vagal motor and other limbic areas through stimulation of the auricular branch of the vagus nerve (Van Laere et al., 2000). Among them, polysynaptic fibers emitted from the solitary tract nucleus project to the reticular formation in the middle of the medulla oblongata, the parabrachial nucleus, the blue spot, the amygdala, the hypothalamus, the frontal–orbital gyrus, the anterior cingulate gyrus, and other structures that are closely related to anxiety and depression and can play a role in regulating sleep and improving the mood of patients with pain. A functional magnetic resonance imaging (fMRI) study revealed that in 22 healthy subjects, taVNS-induced enhancement of mood was accompanied by a significant decrease in activity in subcortical limbic regions, including the amygdala. This finding underscores the importance of taVNS in modulating pain perception (Kraus et al., 2007; Flores et al., 2024). Consequently, it is hypothesized that taVNS may not only regulate patients’ anxiety by modulating negative emotions but also influence their perception of pain, which in turn affects their overall pain ratings.

Among all HRV parameters, the RMSSD and HF have been shown to reflect parasympathetic activity, whereas the SDNN and LF reflect the balance between the sympathetic and parasympathetic nervous systems. Although the effects of taVNS on cardiac autoregulation have been the subject of previous studies, the present study went a step further to determine the effect of taVNS on postoperative pain after bilateral tibiofibular fractures. However, it was not possible to elaborate more precisely on its neurophysiological mechanisms. However, human studies measuring cardiac sympathetic neurotransmitter release suggest that LF reflects blood pressure reflex function (Goldstein et al., 2011). Autonomic interventions in humans have demonstrated that increases in arterial or venous blood pressure are associated with hyperalgesia. When blood pressure increases, signals are transmitted to the brainstem via sympathetic afferent fibers, and the brain, particularly the hypothalamus, reduces systolic blood pressure and total peripheral resistance by regulating sympathetic fiber output in the reflex heart and peripheral vasculature, respectively. Consequently, parasympathetic efferent fiber reflexes are accelerated, leading to a decrease in heart rate and a reduction in blood pressure (Kaniusas et al., 2019). In healthy and normotensive populations, elevated arterial blood pressure at rest also leads to reduced pain sensitivity (Bruehl et al., 1992). Experimental models of acute and chronic hypertension have demonstrated (Bruehl et al., 1992) that disruption of sinus and aortic nerve inputs attenuates or eliminates the hyperalgesia associated with hypertension, in part by augmenting the ascending conduction pathway for pain. Experimental hypertension reduces pain sensitivity by inhibiting pain signaling at the spinal level. However, human experiments in which sympathetic neuroreceptors are activated by cervical suction are impractical in acute surgery. Therefore, the present findings support the hypothesis that autonomic modulation through activation of the parasympathetic nervous system is antagonistic to the sympathetic nervous system and is one of the expected therapeutic mechanisms of taVNS.

In summary, after non-cardiac surgery, taVNS not only modulates the autonomic nervous system to alleviate postoperative pain but also reduces the dosage of analgesic medications, minimizing the impact of postoperative pain on patients’ physical function. Therefore, this study preliminarily demonstrates the analgesic efficacy of taVNS for postoperative pain in bilateral tibiofibular fractures. However, whether it can serve as an effective clinical analgesic method still requires extensive randomized controlled trials for further investigation.

## 5 Compliance and safety

Compliance was assessed by recording the start time, end time, stimulation duration, and intensity for each taVNS session. Records indicated that patients averaged one daily session over the two-week period, with a mean single-session duration of 30 min and intensity maintained at 2.0 ± 0.5 mA. Patients documented stimulation time, duration, left-ear electrode positioning, and abnormal sensations in diaries, showing > 90% concordance with device-recorded data.

During the 12-day intervention, daily follow-up examinations were performed: Auricular skin inspections revealed no erythema, pruritus, or other abnormalities, and patients reported no dizziness, headache, cough, or other systemic adverse reactions. Pre- and post-intervention ECG monitoring ruled out cardiovascular abnormalities. Patients were specifically queried regarding facial asymmetry or mastication weakness to detect accidental stimulation of the facial or trigeminal nerves. No adverse events occurred throughout the intervention period.

## 6 Patient perspective

The patient provided written informed consent for this study. Regarding pain intensity changes, the patient self-reported that their Visual Analog Scale (VAS) score decreased from 8 pre-intervention to 2 post-intervention. Compared to pre-intervention status, the average analgesic consumption also significantly reduced. Following electrical stimulation, continuous nightly sleep duration exceeded 5 h. Additionally, the patient reported enhanced rehabilitation confidence. Throughout the intervention, mild pain and a tingling sensation described as “weak electric current” occurred at the left auricular site but remained tolerable. In summary, the patient tolerated the intervention well, experienced substantial pain reduction, and reported bolstered confidence in recovery.

## 7 Strengths and limitations

This study provides a novel non-pharmacological and non-invasive approach to postoperative pain management by stimulating the auricular branch of the vagus nerve to directly modulate the autonomic nervous system (ANS). This method may reduce the addictive potential and gastrointestinal side effects associated with conventional analgesics to varying degrees. Furthermore, the adoption of heart rate variability (HRV) as an objective biomarker for evaluating pain and ANS dynamics offers advantages in terms of reliability and resistance to subjective manipulation. Finally, a comprehensive assessment was conducted through the integration of subjective and objective pain indicators, combined with psychophysiological correlations.

The present study is subject to certain limitations. First, the duration of the intervention was only 2 weeks, and the analgesic effect beyond this timeframe was not monitored. Second, as this is currently only a case report, the sample size is small, and the statistical significance may be insufficient; therefore, the conclusions lack broad representativeness. The absence of a control group in the case report further complicates the assessment of the treatment effect, as it hinders the ability to distinguish between the treatment effect and any spontaneous recovery. Consequently, a substantial number of randomized controlled trials are needed to validate the findings and ensure the reliability of the results.

## Data Availability

The raw data supporting the conclusions of this article will be made available by the authors, without undue reservation.
